# Typical disease courses of patients with unipolar depressive disorder after in-patient treatments–results of a cluster analysis of the INDDEP project

**DOI:** 10.3389/fpsyt.2023.1081474

**Published:** 2023-04-06

**Authors:** Theresa Martinek, Marc Jarczok, Edit Rottler, Armin Hartmann, Almut Zeeck, Heinz Weiß, Jörn von Wietersheim

**Affiliations:** ^1^Department of Psychosomatic Medicine and Psychotherapy, Ulm University Medical Center, Ulm, Germany; ^2^Department of Psychosomatic Medicine and Psychotherapy, Medical University Hospital, Freiburg, Germany; ^3^Department of Psychosomatic Medicine and Psychotherapy, Robert-Bosch-Krankenhaus, Stuttgart, Germany

**Keywords:** major depression (MD), treatment response groups, follow up examination, cluster analysis, in-patient treatment

## Abstract

**Introduction:**

Previously established categories for the classification of disease courses of unipolar depressive disorder (relapse, remission, recovery, recurrence) are helpful, but insufficient in describing the naturalistic disease courses over time. The intention of the present study was to identify frequent disease courses of depression by means of a cluster analysis.

**Methods:**

For the longitudinal cluster analysis, 555 datasets of patients who participated in the INDDEP (INpatient and Day clinic treatment of DEPression) study, were used. The present study uses data of patients with at least moderate depressive symptoms (major depression) over a follow-up period of 1 year after their in-patient or day-care treatments using the LIFE (Longitudinal Interval Follow-Up Evaluation)-interview. Eight German psychosomatic hospitals participated in this naturalistic observational study.

**Results:**

Considering only the Calinski–Harabatz index, a 2-cluster solution gives the best statistical results. In combination with other indices and clinical interpretations, the 5-cluster solution seems to be the most interesting. The cluster sizes are large enough and numerically balanced. The KML-cluster analyses revealed five well interpretable disease course clusters over the follow-up period: “sustained treatment response” (*N* = 202, 36.4% of the patients), “recurrence” (*N* = 80, 14.4%), “persisting relapse” (*N* = 115, 20.7%), “temporary relapse” (*N* = 95, 17.1%), and remission (*N* = 63, 11.4%).

**Conclusion:**

The disease courses of many patients diagnosed with a unipolar depression do not match with the historically developed categories such as relapse, remission, and recovery. Given this context, the introduction of disease course trajectories seems helpful. These findings may promote the implementation of new therapy options, adapted to the disease courses.

## 1. Introduction

Depressive disorders affect approximately 322 million people worldwide (1). This number has continuously increased over the last years. From 2007 to 2017 a worldwide increase of 14.3% and from 1990 to 2017 a global increase of 334% was observed (2). These numbers highlight, that depressive disorders constitute a global problem with the tendency to exacerbate. Simultaneously, in many cases the disease course is of a long-term nature with periodical relapses. This stresses the importance to identify clusters in long-term depression courses, firstly to improve interventions (3), and secondly to enhance the prediction of long-term disease courses in individual patients.

The symptom changes that occur during or after the treatment of a (recurring) unipolar depression can be summarized in different categories to allow a classification in accordance with the German S3 guidelines (4). Given this context, it is important to stress the differentiation between the concepts of relapse and recurrence. A relapse is defined as the reappearance of an illness within 12 months after a previous improvement of the symptoms to a subclinical level. A recurrence however, refers to the reappearance of a new episode of the disease after a previous remission of the symptoms for at least 12 months (4). A remission is defined as “full recovery of the previous functional state or an extensively symptom free state after an acute therapy” (4), while a response is considered a “reduction of the depression scores in pertinent questionnaires (e.g., BDI, PHQ-D, HDRS) of at least 50% as compared to the initial assessment.” Analogue, the Australian guidelines (RANZCP) define the categories of treatment response, remission, full recovery and recurrence with regard to a unipolar depression (5).

Concluding, the presented and widely accepted classification of the symptom changes by the S3 guidelines of the DGPPN highlight the extensive variability in the development of depressive disorders throughout the different treatment phases. However, it is important to highlight that these definitions do not aim to describe naturalistic disease courses over time. They were primarily developed as clinically relevant outcome criteria for the evaluation of (pharmacological) treatments (6). Historically, the MacArthur Foundation Research Network on the Psychobiology of Depression discovered “serious inconsistencies and problems in defining change points in the clinical course of depression” (Monroe and Harkness, p. 658) (7), which led to the creation of a task force in 1988 to investigate the matter. Frank et al. (6) were firm about the provisional nature of the proposed conceptual scheme and “enthusiastically invited others to challenge [their] tentative suggestions with alternative conceptualizations and for empirically derived criteria” (Frank et al., p. 855).

Following this suggestion, the main goal of the “INDDEP” (INpatient and Day clinic treatment of DEPression) -study was to find prognostic and prescriptive predictors for the differential diagnostic evaluation and indication, as well as the clinical course (8–11). The variability of the courses in this study was analyzed at four assessment time points (in-patient admission, hospital discharge, 3-months follow-up, 12-months follow-up) (12).

**FIGURE 1:**
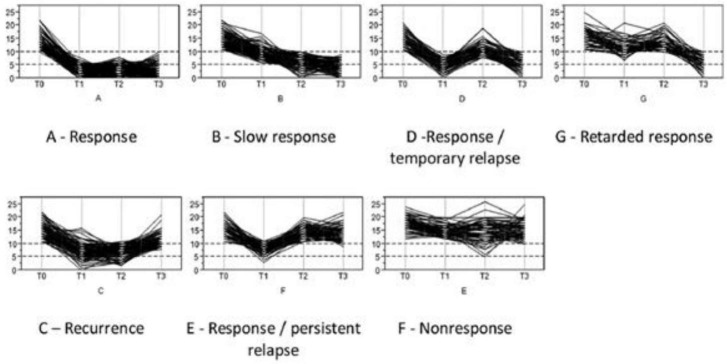
The KML-cluster analysis yielded seven depression courses; patients of the INDDEP-study, eight study centers in Germany, 2011–2015 [reused from: (12). Reproduction approved by Elsevier publishing, license number: 5272891211810].

Hartmann et al. (12) analyzed the disease courses in the same study (INDDEP) using the QIDS (Quick Inventory for Depressive Symptomatology) expert rating (13) at the four timepoints. The performed KML-cluster analysis (14, 15) yielded the best result for a solution with seven clusters. The resulting clusters show the disease courses: “response,” “slow response,” response/temporary relapse,” “delayed response,” “recurrence,” “response/persistent relapse,” “non-response” (12). The listed courses are illustrated in Figure 1.

The main question of the present study was to analyze if the weekly depression values as assessed in the follow-up interviews resulted in different clusters or if the clusters as identified at the four timepoints of measurement correspond to the clusters found by Hartmann et al. (12). Source of the analysis are the weekly depression values of the patients of the INDDEP-study as assessed with the Longitudinal Interval Follow-Up Evaluation (LIFE) -interviews during the 1-year follow-up period.

## 2. Materials and methods

### 2.1. Study design

The data used in this study stem from the multicentric “INDDEP”- Study, which was approved by the ethics committees of Ulm University and Freiburg University (39/11 and 83/11). The study was registered in the ISRCTN (20317064). Within the frame of this naturalistic observational study, data of patients with at least one moderately expressed depressive disorder were assessed at eight German psychosomatic centers with in-patient and day hospital departments. Recruitment of the patients occurred over a period of 36 months from 2011 to 2014. Data collection took place at four timepoints for every participating patient (T0-T3): at admission (T0) and discharge from the in-patient or day hospital (T1), as well as 3- (T2) and 12 (T3) months after hospital discharge. At T0 and T1 data acquisition took place on-site at the respective hospital where the treatment occurred. At T2 and T3 trained interviewers of the universities of Ulm and Freiburg conducted interviews *via* telephone, which allowed data collection of the disease course. Additionally, patients received postal questionnaires, which they were asked to fill out and return to the study’s main office. The interview at T2 gathered retrospective data over the course of the past 3 months after discharge. At T3, identical interviews and questionnaires were used, retrospectively assessing the period from T2 to T3, thus referring to a period of approximately three to 12 months after discharge. To assess the pathological severity the LIFE interview (described below) was used.

### 2.2. Sample

A total of 604 patients participated in the INDDEP study. Main inclusion criterion was the presence of at least one moderate depressive disorder in accordance with the ICD-10. This was defined using the QIDS-C-questionnaire (Quick-Inventory of Depressive Symptomatology), with a cut-off value of at least 10. Furthermore, only patients with a main diagnosis in line with the ICD-diagnoses F32 or F33 (depressive episode or recurring depressive disorder) were included. All patients received treatment for their depressive disorder, either in an in-patient setting or a day hospital. Age ranged from 19 to 65 and all were fluent in German. Patients with a comorbid disorder, such as antisocial personality disorder, current or previous psychotic episodes, bipolar disorder, substance dependency, or present suicidality, were excluded. Cognitive impairments like dementia lead to exclusion. More information on the INDDEP sample and screening for participation can be found in Zeeck et al. (9, 10).

A total of 49 participants were excluded due to missing follow-up data. Thus, 555 records from the LIFE interviews served as the sample for the analyses. The sample consists of 360 women (65%) and 195 men (35%). Age ranged from 19 to 65 years (mean = 43, SD = 11.7). The mean duration of hospital treatment was 10 weeks (SD = 4.3 weeks). During the follow-up period, 80% of the patients received psychotherapy and 67% received antidepressant medication. Further details of the follow-up treatments are described in Weiss et al. (8).

### 2.3. Longitudinal interval follow-up evaluation (LIFE-interview)

The LIFE-interview is an assessment instrument to evaluate the long-term course of psychiatric disorders (16). It includes an instruction, a semi-structured interview, a coding sheet, as well as multiple training materials. At follow-up intervals, various psychosocial and psychopathological details can be investigated, as well as additional treatments. Psychopathology is assessed by psychiatric status ratings (PSR) with symptom based ordinal scales (16), which have been found to have good to excellent interrater reliability (17–19).

Since the interview was conducted at two follow-up timepoints [T2 and T3, thus three and 12 months after discharge (T1)], we were able to document a period of approximately 1 year. The LIFE-interview was conducted to assess the symptoms retrospectively for every week and thus to evaluate the course of the depression during the follow-up period. The interviews were conducted on the phone. Four interviewers from the universities of Ulm and Freiburg had received extensive training in interviewing techniques and coding of the LIFE. The coding of the depression severity occurred using the 6-point Major Depression Episode- (MDE) scale. The healthy state (without any residual symptoms) is coded with MDE 1, mild residual symptoms with MDE 2, partial remission with 3, significant depressive symptoms with 4, meeting of the criteria of a depressive disorder with 5, and additional psychotic symptoms or extreme impairments with MDE 6. Thus, higher values represent an increased severity of the measured variable “depression.” To remember the depression severity for each week, patients were asked to orientate themselves using anchor data (e.g., Christmas, birthdays). Additional data e.g., the current medication or psychotherapeutic treatments were assessed using the LIFE-interview.

### 2.4. Data analysis and statistics

For the analyses of the disease courses during the follow-up period, 52 weekly depression scores per patient were imported into the statistical programme R-studio (version 3.6.3). However, the KML cluster analysis over the 52 time points required a reduction in the number of categories used. For this purpose, clinically related MDE scores of the psychiatric status assessment were combined and recoded into four severity categories. Severity 1 includes patients in a healthy state (no residual symptoms), corresponding to an MDE score of 1. Severity 2 includes patients with MDE scores of 2 and 3, i.e., mild residual symptoms and partial remission. Severity 3 includes patients with an MDE score of 4, i.e., with significant depressive symptoms. Severity 4 includes patients who meet all criteria for a depressive episode, including possible psychotic symptoms or extreme impairment (MDE scores 5 and 6).

A KML cluster analysis for longitudinal data (14, 15) was performed to calculate the different disease trajectories during the follow-up period. This was done using the R statistical program (version 4.2.1, default KML settings). As a variant of k-means cluster analysis, it is specifically designed for the analysis of dependent measures. However, a common problem is determining the appropriate number of clusters. In general, KML offers five different standardized indices for selecting the “best” number of clusters (15). These five criteria are non-parametric and can be calculated without making any hypotheses (15). As the main criterion, the Calinski–Harabasz index (20) is used when calculating a KML cluster analysis (14) (also known as the Variance Ratio Criterion). It is calculated as the ratio between the sum of the inter-cluster dispersion and the sum of the intra-cluster dispersion for all clusters. The optimal number of clusters is obtained with the highest Calinski–Harabasz score (15). In a comparative study, the Calinski–Harabasz index was found to be superior to other indices in detecting the “best” number of clusters (21). However, clinical criteria should be considered when deciding which cluster to use: The cluster should discriminate between remission and relapse, and the number of participants in the clusters should be large enough; furthermore, there should not be too many clusters, and additional clusters should provide meaningful clinical information.

For the most (clinically) interesting cluster solution, important data of the patients in the different clusters were compared, using an ANOVA or Chi2-Tests. Data like gender, age, illness duration, duration of sick-leave, QIDS depression scores at intake and discharge were compared between the members of the cluster.

## 3. Results

In order to graphically represent the different disease courses of the patients’ depressive disorders over the given weeks, the KML cluster analysis was calculated and interpreted for different numbers of clusters. In the analysis, the Calinski–Harabasz index decreased consistently with increasing number of clusters (363.46 for the two-cluster solution and 153.30 for the seven-cluster solution). Accordingly, the 2-cluster solution gives the statistically best results, followed by the 3-cluster solution. A consistency check with the Ray and Touri and Davies and Bouldin indices gives the same result. In addition, taking these indices into account, the 5-cluster solution must be considered as the statistically third best solution. The three statistically superior cluster solutions are presented below. For more information on the other cluster solutions (up to 7 clusters), please see the Supplementary material 1.

### 3.1. 2-cluster solution

**FIGURE 2:**
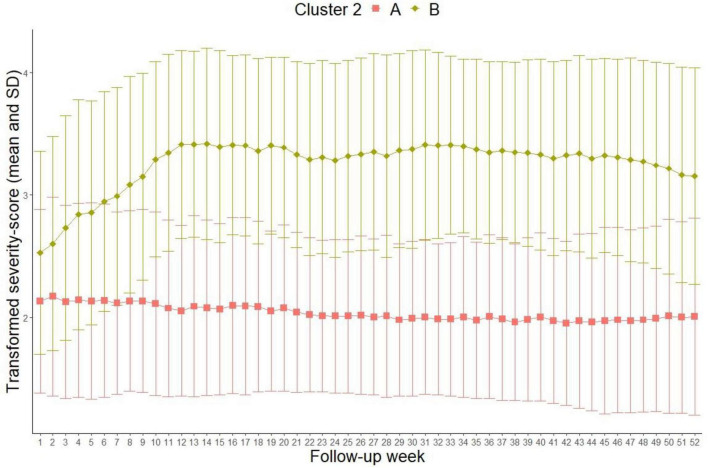
2-cluster-solution (Transformed severity score out of MDE 6-point scale: 1 = 1 in MDE 6-point scale = healthy, 2 = 2 and 3 in MDE 6-point scale = residual symptoms and partial remission, 3 = 4 in MDE 6-point scale = depressive symptomatology without meeting full diagnostic criteria, 4 = 5 and 6 in MDE 6-point scale = meeting diagnostic criteria for unipolar depression and showing possible psychotic symptoms or extreme impairment).

Figure 2 illustrates the results of the cluster analysis with two clusters (Calinski–Harabasz index 363.5).

The mean depression score of patients in cluster “A” (*N* = 344, 62%) is mostly constant with an average severity score of approximately 2. For patients in cluster “B” (*N* = 211, 38%), the average severity score at discharge (follow-up week 1) was 2.5. Until week 10 the average increases to 3.5 and stays relatively constant. Clinically, this means that patients in cluster “A” present residual symptoms or a partial remission during the entire follow-up period. After the inpatient treatment, patients in cluster “B” initially present residual symptoms and marked depression symptoms. However, a worsening of the symptomatology manifests from week 10 to the end of the follow-up period in the direction of a full-syndrome disorder. Thus, the disease courses in cluster “A” can be referred to as sustained treatment response, while patients in cluster “B” present a persistent relapse.

### 3.2. 3-cluster solution

**FIGURE 3:**
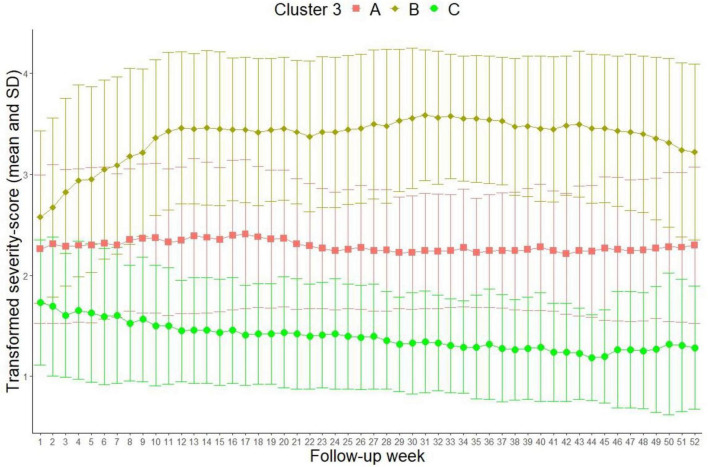
3-cluster solution (Transformed severity score out of MDE 6-point scale: 1 = 1 in MDE 6-point scale = healthy, 2 = 2 and 3 in MDE 6-point scale = residual symptoms and partial remission, 3 = 4 in MDE 6-point scale = depressive symptomatology without meeting full diagnostic criteria, 4 = 5 and 6 in MDE 6-point scale = meeting diagnostic criteria for unipolar depression and showing possible psychotic symptoms or extreme impairment).

Figure 3 illustrates the cluster analysis with three clusters (Calinski–Harabasz index 263.7).

The mean depression values in cluster “A” (*N* = 300, 54.1%) and “B” (*N* = 176, 31.7%) are largely unvaried to the solution with two clusters (clinical description see above). In the new cluster “C” (*N* = 79, 14.2%), the mean depression scores are low at discharge (1.75) and continually decrease during the follow-up period. Toward the end, the patients approach a mean depression value of 1, which is comparable to a state without residual symptoms. Clinically, cluster “C” represents a remission.

### 3.3. 5-cluster solution

**FIGURE 4:**
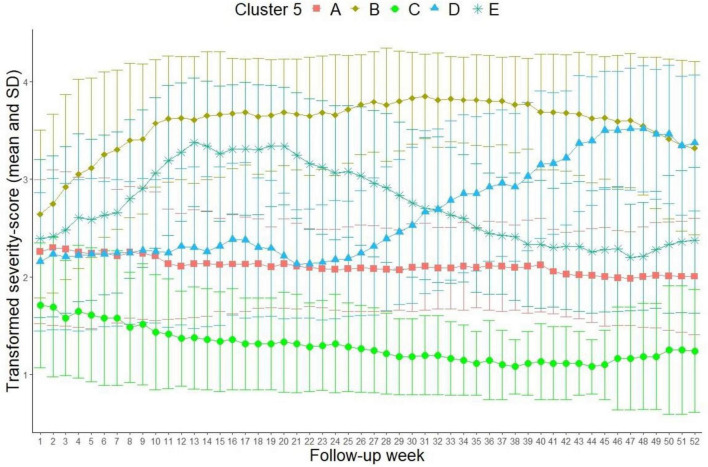
5-cluster solution (Transformed severity score out of LIFE MDE 6-point scale: 1 = 1 in MDE 6-point scale = healthy, 2 = 2 and 3 in MDE 6-point scale = residual symptoms and partial remission, 3 = 4 in MDE 6-point scale = depressive symptomatology without meeting full diagnostic criteria, 4 = 5 and 6 in MDE 6-point scale = meeting diagnostic criteria for unipolar depression and showing possible psychotic symptoms or extreme impairment).

The Calinski–Harabasz index was 190.4 for the 5-cluster solution (Figure 4).

The mean depression values of the patients in cluster “A” (*N* = 202, 36.4%), “B” (*N* = 115, 20.7%) and “C” (*N* = 63, 11.4%) are similar to the 3-cluster solution. New in these results are cluster “E” (*N* = 95, 17.1%), and “D” (*N* = 80, 14.4%). In cluster “E,” the mean depression values of the patients increase from 2.4 to 3.4 over the course of the first 15 follow-up weeks and then decrease to a level of 2 (partial remission) by the end of the follow-up period in week 52. From a clinical point of view, these patients present a temporary relapse, even though the magnitude of the symptomatology varies from residual symptoms to considerable main symptoms of a depressive episode. In cluster “D” however, the course of the depression is almost parallel to cluster “A” until week 25. The mean depression values in cluster “D” increase to approximately 3.5, which is a clinical recurrence, beginning approximately in week 25.

Additional comparisons between the members in these five clusters showed, that age, gender and the number of patients with a first depressive episode compared to those with more than one episode did not differ significantly between the clusters. Patients in the problematic clusters B and D had significantly higher depression scores at admission and discharge. They took longer sick leaves before the treatments and had more depressive episodes before treatment. Patients in Cluster D had significantly longer illness durations than the others. The tables of these comparisons are shown in Supplementary material 2.

## 4. Discussion

The main question of the present study was related to the clusters of the weekly depression scores during the follow-up period of patients who participated in the INDDEP-study. The KML-cluster analysis with two clusters presented the highest Calinski–Harabasz index and is statistically the most effective cluster formation. At the same time, clinical interpretability and explanatory power need to be taken into account, which is clearly limited when using only two clusters. By linking the above listed clinical criteria, a partition with five clusters could be identified as the most interesting solution. This partition is clinically meaningful and generates clusters, which are large enough and numerically well balanced. The identified five clusters depict the following different paths of depression courses: Cluster “A” describes the course of the disorder with a sustained treatment response, while the patients in cluster “B” suffer a persistent relapse. Cluster “C” represents all the patients with a temporary relapse, while cluster “D” and “E” incorporate patients with a recurrence or remission, respectively.

Our cluster solutions assessing weekly follow ups cannot replicate the results of Hartmann et al. (12), who also used data of the INDDEP study. However, the identified clusters correspond to a great extent: the courses classified as “sustained response,” “recurrence,” “persistent relapse,” and “temporary relapse” can be found in both works. Additionally, Hartmann et al. (12) identified courses with a “slow response” and “delayed response” and “non-response.” An explanation for the deviation can be found in the different analyzed time periods. The present work analyzed weekly depression values during the follow-up period after hospital treatment as assessed with the LIFE-interview. Hartmann et al. (12) used the QIDS scores to assess the depression values at four timepoints (admission, discharge, three-, and 12 months follow-up). Next, it is important to consider the well-known problems of one-dimensionality and longitudinal measurement invariance in different rating scales of depression (22), which can, to a certain extent, explain the different results.

Others studies that investigated the disease course of depressive disorders showed inconsistent results regarding the number of clusters and the identified trajectories. Another multicenter observation study (23) assessing depressed patients equivalent to the INDDEP study, found seven different clusters of depression courses, analogue to the results of Hartmann et al. (12). A longitudinal study, which conducted a latent class analysis identified nine different clusters (24). These nine clusters can be grouped into three courses of poor treatment responses, three courses of an early treatment response and three courses with a late treatment response (24). A study with depressed adolescents aged 11 to 17 years in England presented only two disease trajectories: a continuous improvement and an interrupted improvement (25). Another study assessing patients with major depression receiving antidepressant medication also identified two disease trajectories: responders (76.3%) and non-responders (23.7%) (26). Similar to our study, a clinical study with depressed patients in the USA identified five different disease courses: early remission, late remission, gradual remission, no treatment response and minimal treatment response (27).

Causes of the differences in the various studies can be found in the different number of timepoints of measurement and in the varying observation periods, as well as in the inconsistently assessed symptom severity. Furthermore, the treatments of the depressed patients in the studies, as well as the different forms of cluster analyses seem to explain the variability of the results to a certain degree. The presented heterogeneity in the results of longitudinal courses can also be found in studies addressing the trajectories of depressive symptoms in the general population: A systematic review summarizing 25 studies found trajectories ranging from three to six clusters (28).

The following strengths and limitations should be mentioned: Firstly, the INDDEP study is a multicenter, naturalistic and prospective observational study. The data from eight German psychosomatic hospitals yield a comparatively large number of participants from rural and urban areas in Germany, which allows some generalizability of the results. On the other hand, the results of the present study can only serve for conclusions for depressed patients, who participated in the treatment of a psychosomatic hospital. Patients with a severe depression and possible apathy and lack of energy, who would have been overstrained with the treatment programme in a psychosomatic clinic, were not included in the INDDEP-study. Also, treatment compliance and treatment options in the health care system may affect the outcome.

Another weakness of the methodology is the fact that follow-up data could only be gathered at two timepoints after 3 months (T2) and after 12 months (T3). Data were assessed by trained interviewers *via* telephone using the standardized LIFE interview. However, the data of the LIFE-interviews were assessed retrospectively and are prone to a recall bias. The patients had to retrospectively remember the respective severity of their depression over large periods of time (up to 40 weeks between T2 and T3; respectively 15 weeks between T1 and T2). The interviewers reported that the assessment of these long periods was sometimes strenuous. However, the LIFE-interview is considered the gold standard for the observation of longitudinal courses of psychiatric disorders and has globally been used in many epidemiological and clinical studies (29).

Another limitation was found in the cluster analysis. Since cluster analyses of longitudinal data are still in development, future advances may offer enhanced clustering methods. Given this context, we would like to highlight that there is currently no scientific consensus regarding the appropriate number of clusters (12) and a KML cluster analysis cannot deliver correct and precise information regarding the “right” number of clusters (14, 15).

Future research should focus on the implementation of disease trajectories which are not covered by the historically established concepts of outcomes (30). This seems particularly important for the development of adapted treatment options.

## Data availability statement

The raw data supporting the conclusions of this article will be made available by the authors, without undue reservation.

## Ethics statement

The studies involving human participants were reviewed and approved by the Ethics Committees of Freiburg University and Ulm University. The patients/participants provided their written informed consent to participate in this study.

## Author contributions

TM and JW wrote the manuscript. AZ was the head major researcher of the INDDEP study. AH, MJ, and ER were responsible for the statistical analyses and performed them. HW belongs to the directory board of the study. The INDDEP Study Group consists of the directors and researchers of the different sites in this study. All authors contributed to the article and approved the submitted version.
