# Supplementary material for: Typical disease courses of patients with unipolar depressive disorder after in-patient treatments–results of a cluster analysis of the INDDEP project
Source: Front Psychiatry. 2023 Apr 6;14:1081474. doi: 10.3389/fpsyt.2023.1081474 (PMC10117975; doi:10.3389/fpsyt.2023.1081474)

Supplement 1: Additional Cluster Solutions and Quality Criteria

Tab. 1: Distribution of cluster sizes in percent (N=555)

	A	B	C	D	E	F	G
2 Clusters	62.0%	38.0%					
3 Clusters	54.1%	31.7%	14.2%				
4 Clusters	43.4%	22.2%	13.2%	21.2%			
5 Clusters	36.4%	20.7%	11.4%	14.4%	17.1%		
6 Clusters	30.5%	16.9%	11.2%	13.7%	16.2%	11.5%	
7 Clusters	28.5%	14.4%	10.1%	14.4%	11.0%	10.8%	10.8%

Figure 1: Quality criteria from KML analyses

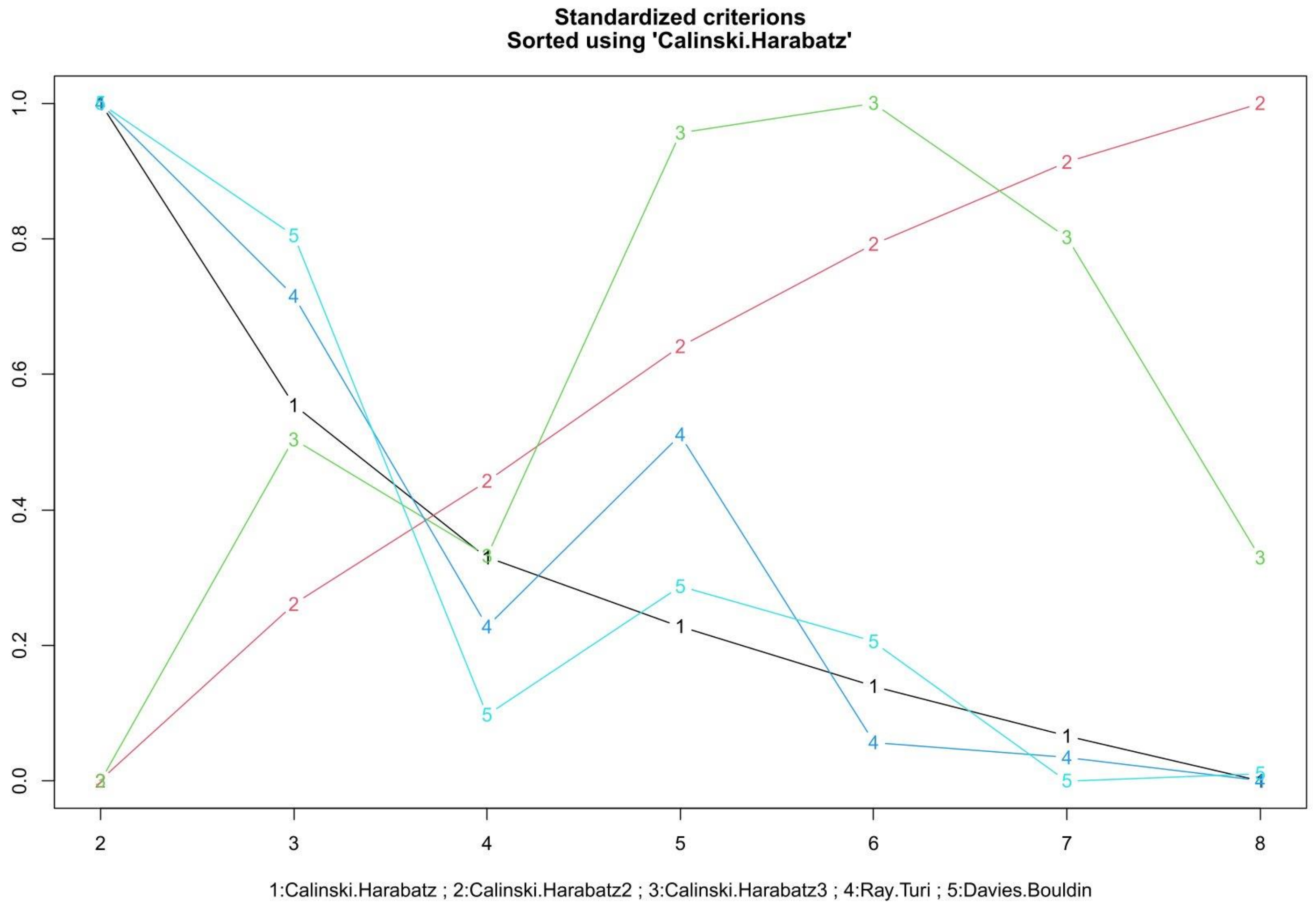

The description of these criteria can be found in Genolini, C., Alacoque, X., Sentenac, M., Arnaud, C. kml and kml 3D: R packages to cluster longitudinal data. *Journal of Statistical Software* (2015) 65: 1-34 (quality criteria on pages 9-11).

Fig. 2: 4-cluster solution (Calinski-Harabatz-Index 212.1)

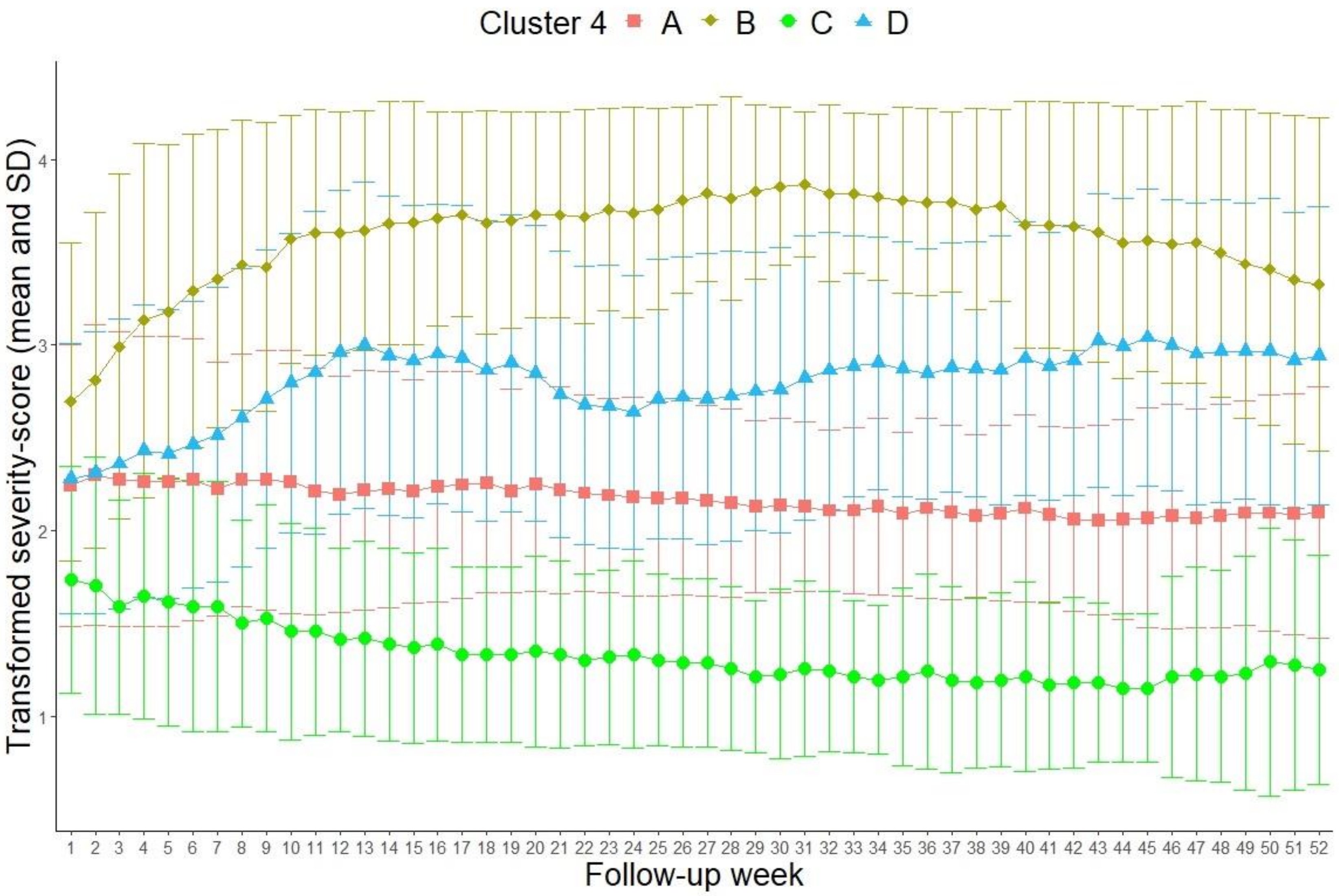

Figure 3: 6-cluster solution (Calinski-Harabatz-Index 171.1)

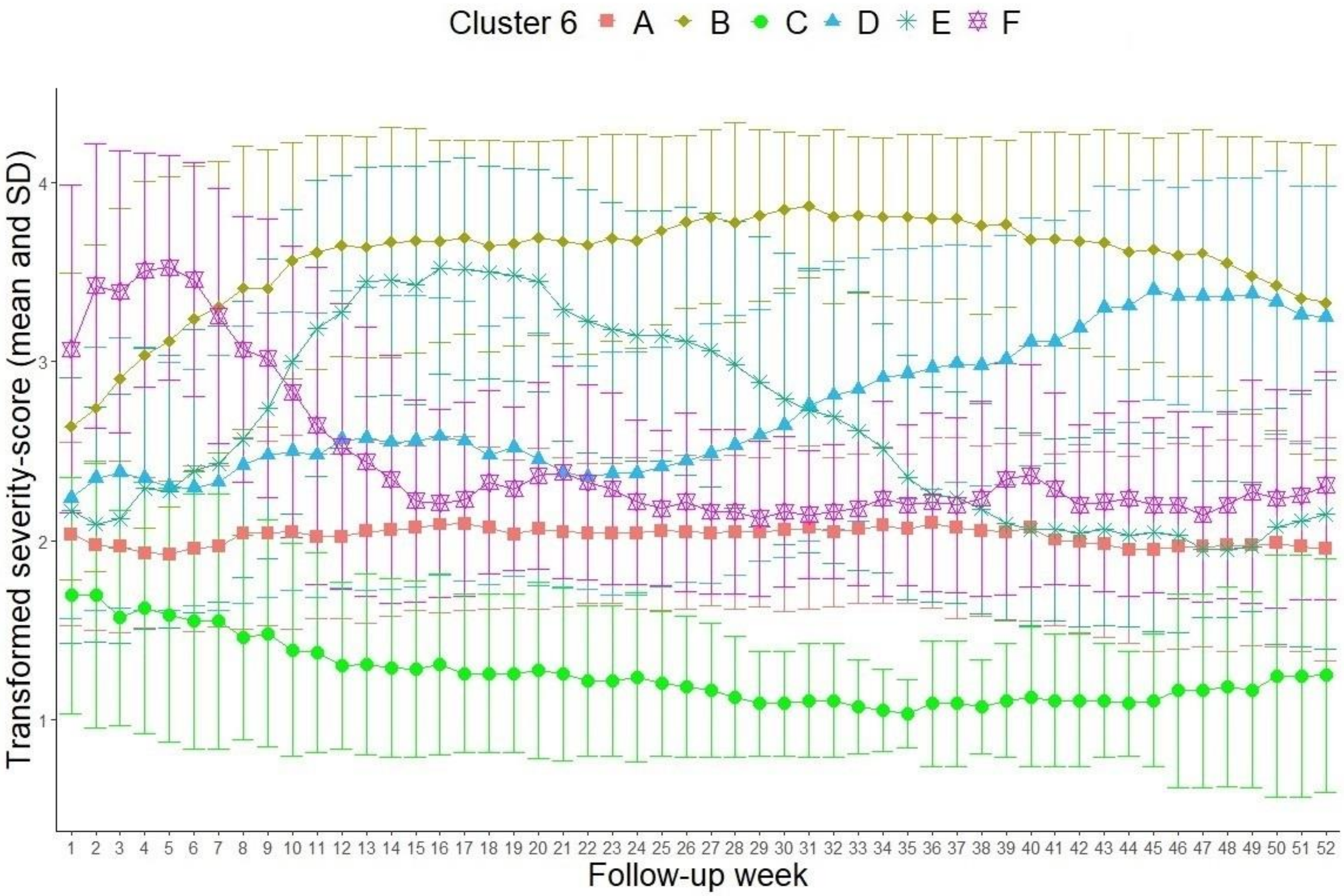

Figure 4: 7-cluster solution (Calinski-Harabatz-Index 153.3)

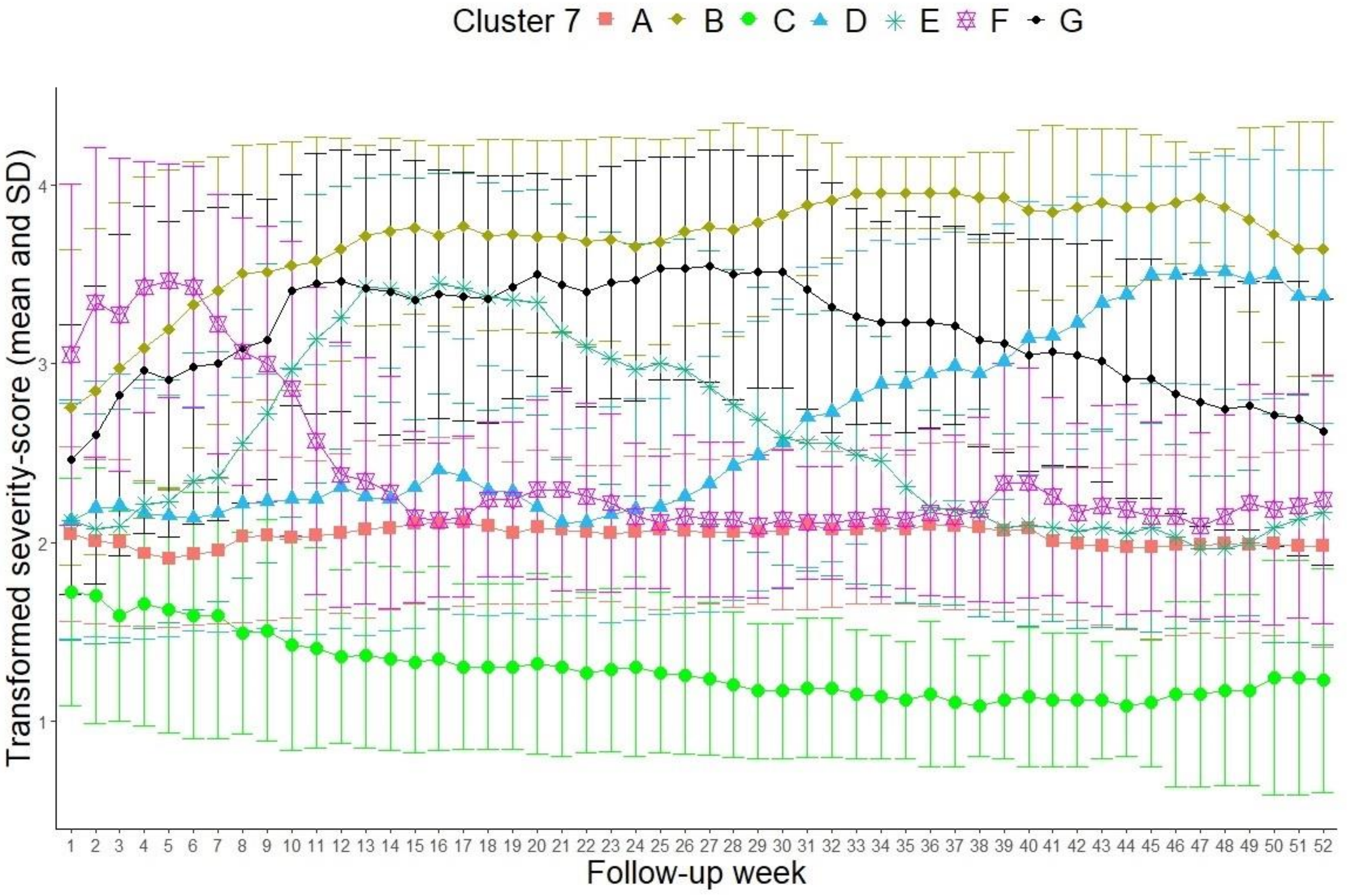

Supplement: Supplementary file 1 [file Data_Sheet_1.pdf]
