# Supplementary material for: Typical disease courses of patients with unipolar depressive disorder after in-patient treatments–results of a cluster analysis of the INDDEP project
Source: Front Psychiatry. 2023 Apr 6;14:1081474. doi: 10.3389/fpsyt.2023.1081474 (PMC10117975; doi:10.3389/fpsyt.2023.1081474)
Supplement: Supplementary file 2 [file Data_Sheet_2.pdf]

Supplement 2: Group comparisons in the 5-cluster solution

Additional comparisons between the members in these five clusters showed, that age, gender and the number of patients with a first depressive episode compared to those with more than one episode did not differ significantly between the clusters. Patients in the problematic clusters B and D had significantly higher depression scores at admission and discharge. They had longer sick leaves before the treatments, and had more depressive episodes before treatment. Patients in Cluster D had a significantly longer illness duration than the others.

Tab. 1: Gender and 5 cluster solution

		Clusters					
		A	B	C	D	E	Total
Gender	male	63	42	26	30	34	195
	female	139	73	37	50	61	360
Total		202	115	63	80	95	555

Pearson-Chi-Square	2.732	df=4	p=.604

Tab. 2: First depressive episode, more than one episode and 5 cluster solution

		Clusters5					
		A	B	C	D	E	Total
First depressive episode	1.00	77	31	22	21	30	181
Not the first episode	2.00	122	78	41	56	60	357
Total		199	109	63	77	90	538

Pearson-Chi-Square	5.045[a]	df=4	p=.283

Tab. 3: Number of depressive episodes and 5 cluster solution

		Clusters5					
		A	B	C	D	E	Total
Number of depressive episodes	1.00	77	31	22	21	30	181
	2.00	48	23	25	14	27	137
	3.00	35	23	6	15	10	89
	4.00	14	12	5	11	9	51
	5.00 and more	25	20	5	16	14	80
Total		199	109	63	77	90	538

Pearson-Chi-Square	26.076[a]	df=16	p=.053

Tab. 4: Age, duration of illness, sick leave and 5 cluster solution

	Cluster A (N = 202)		Cluster B (N = 115)		Cluster C (N = 63)		Cluster D (N = 80)		Cluster E (N = 95)				
	M	SD	M	SD	M	SD	M	SD	M	SD	F (Anova)	p (Anova)	Scheffe-Tests (sign. differences)
Age	43.1	12.7	44.5	11.0	43.5	10.8	45.0	11.7	41.5	11.0	1.3	n.s.	
Duration of illness (years)	9.2	11.2	10.4	10.8	9.2	10.9	14.7	14.6	11.9	11.4	3.55	.007	A<D
Sick leave last year in weeks	13.5	13.1	32.2	46.8	13.3	14.0	25.0	25.8	30.2	30.9	7.0	<.001	A<B,E C<B

Tab. 5: QIDS self-ratings and 5 cluster solution

	Cluster A (N = 202)		Cluster B (N = 115)		Cluster C (N = 63)		Cluster D (N = 80)		Cluster E (N = 95)				
	M	SD	M	SD	M	SD	M	SD	M	SD	F (Anova)	p (Anova)	Scheffe-Tests (sign. differences)
QIDS-T0 (self-report)	14,6	4,3	17,7	4,3	13,6	3,8	15,7	4,9	15,2	4,3	12,2	<.001	A,C,D,E<B
QIDS-T1 (self-report)	9.2	4.9	13.7	5.1	5.8	3.9	10.2	5.4	11.7	4.7	33.0	<.001	C<A,B,D,E D<B, A<E
QIDS-T3 (self-report)	8.0	4.1	15.3	5.5	4.4	3.8	14.4	5.4	11.2	4.8	75.9	<.001	C<A<B,D,E E<D<B
